# The details of past actions on a smartphone touchscreen are reflected by intrinsic sensorimotor dynamics

**DOI:** 10.1038/s41746-017-0011-3

**Published:** 2018-03-07

**Authors:** Myriam Balerna, Arko Ghosh

**Affiliations:** 1 Institute of Neuroinformatics, University of Zurich and ETH Zurich, Zurich, Switzerland; 2 Institute of Psychology, Cognitive Psychology Unit, Leiden University, Leiden, The Netherlands

**Keywords:** Sensorimotor processing, Neurology

## Abstract

Unconstrained day-to-day activities are difficult to quantify and how the corresponding movements shape the brain remain unclear. Here, we recorded all touchscreen smartphone interactions at a sub-second precision and show that the unconstrained day-to-day behavior captured on the phone reflects in the simple sensorimotor computations measured in the laboratory. The behavioral diversity on the phone, the speed of interactions, the amount of social & non-social interactions, all uniquely influenced the trial-to-trial motor variability used to measure the amount of intrinsic neuronal noise. Surprisingly, both the motor performance and the early somatosensory cortical signals (assessed using EEG in passive conditions) became noisier with increased social interactions. Inter-individual differences in how people use the smartphone can help thus decompose the structure of low-level sensorimotor computations.

## Introduction

In elite musicians and athletes, cortical sensorimotor processing is associated to the occupation-specific behavioral differences.^1^ Based on the behavior roughly quantified by using questionnaires and diary entries, the sensorimotor cortex reflects the amount of use of the corresponding body part.^2^ This pattern of result may extend to the unconstrained day-to-day activities, as suggested by the correlation between the amplitude of somatosensory cortical potential evoked from the thumb and the amount of smartphone use—roughly estimated by using battery logs.^3^ By further leveraging the technology built into the phone here we address how the details of the behavior—in terms of the temporal pattern and the behavioral context—influence the neural processes underlying sensorimotor processing. Our findings show that the details of the past behavior captured on the phone are associated with the low-level cortical sensorimotor processes.

## Results

We directly logged smartphone behavior, recording all of the touchscreen interactions in 57 participants. After collecting 21 days of data, we tested these participants on a simple reaction time task. In a separate experimental session, we measured the somatosensory cortex as it received afferent inputs from the thumb. In these laboratory tests, we focused on the elementary property of neuronal variability, or ‘‘noise’’, in the sensorimotor system. This property is malleable throughout the life span in the sense that the noise is high through development, diminishes into adulthood and increases again in aging.^4^ Furthermore, substantial theoretical and empirical support exists for the idea that increased use of a body part—as in deliberate practice—reduces sensorimotor noise.^5^

We labeled the Apps as ‘‘Social’’ (such as towards Twitter, Facebook, and WhatsApp) and ‘‘Non-Social’’ (such as Weather and Google Search) as social activity draws more attention, involves longer stretches of rapid finger movements for text messaging, and is associated with neuroplasticity promoting neuromodulation.^6^ The number of touches on Social Apps was only partly correlated with the number of touches on Non-social Apps (variables Log_10_ normalized, *R*^2^ = 0.29, *f* (1,55) = 22, *p* = 1.9 × 10^−6^, robust linear regression). Interestingly, the number of Apps used and the number of touches (on Log_10_ scale) showed little day-to-day variation compared to the typical rate of touchscreen interactions (see Supplementary Fig. 1 & Supplementary Note for detailed description of touchscreen behavior).

**Fig. 1:**
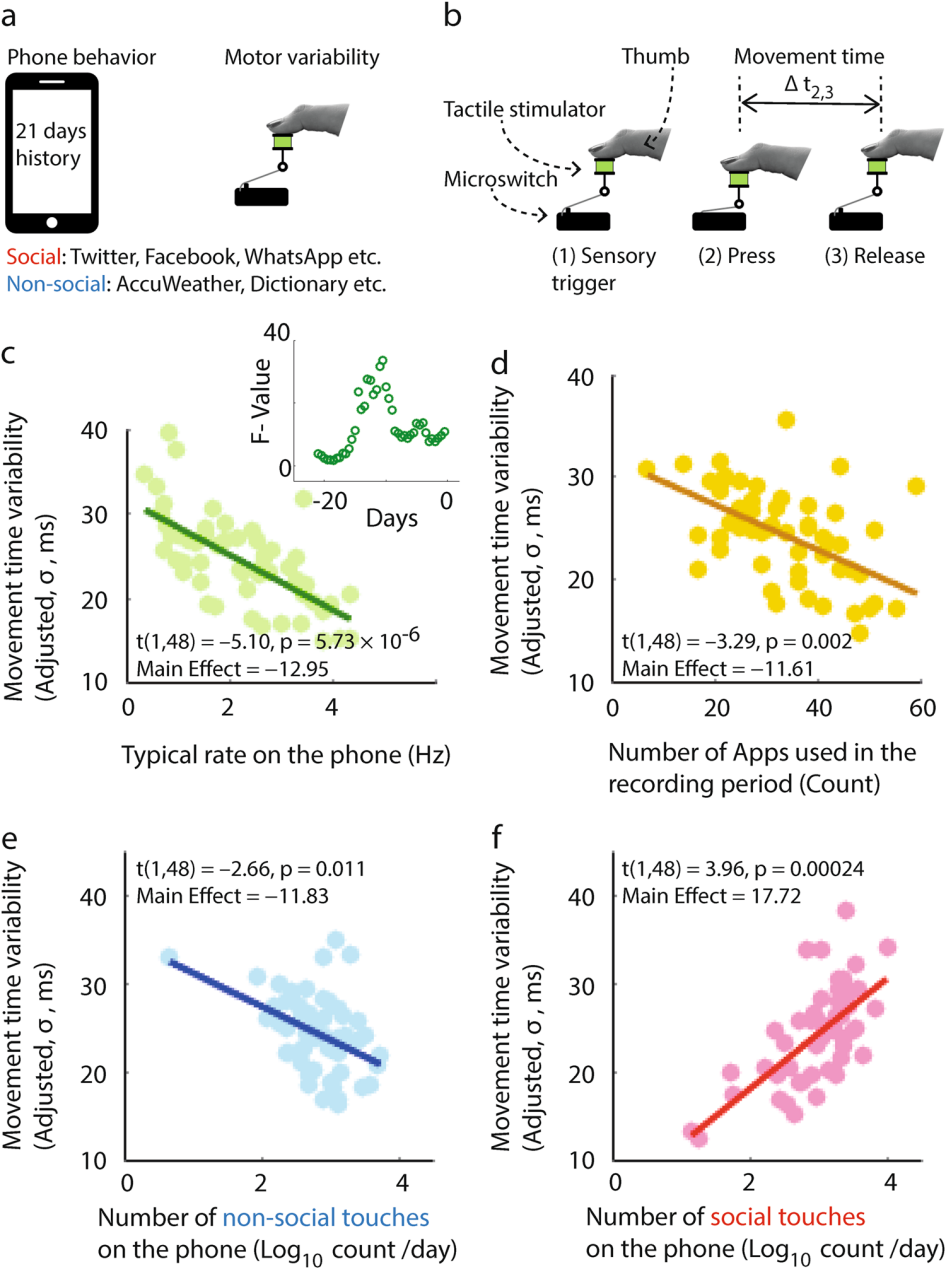
The history of unconstrained touchscreen behavior reflects on the performance of a simple sensorimotor task. **a** Touchscreen activity was recorded for 21 days and followed by laboratory measurements of sensorimotor variability. **b** The task required responding to tactile stimuli by pressing and releasing a micro switch, as fast as possible, with the thumb. *Reaction time* is the time from the sensory stimulus to the press-down action and *movement time* from the pressed position to the release. **c–f** Adjusted response plots. Movement time variability (*σ*) was inversely proportional to the typical rate at which the touchscreen was used (**c**) the number of Apps used (**d**) and the amount of activity on Non-social Apps (**e**). The variability was directly proportional to the amount of activity on Social Apps (**f**). Linear regression statistics is imprinted on the figures. Insert in figure **c** shows how the strength of the relationship with the typical rate varies as a function of the data collection period [assessed by using a 72 h sliding window, sliding with 12 h steps] and that the data collected 10.5 days prior to the experiment yielded the strongest correlation

At the end of the touchscreen recording period, the participants performed a simple tactile reaction task in the laboratory, which involved micro switch press-down and release-up actions (Fig. 1a, b). In theory, the time taken to trigger the press-down action (reaction time), captures the sensory decision processes, and the time taken to complete the motor act, from pressing down to releasing upwards (movement time), captures the lower cognitive levels of sensorimotor execution.^7,8^ Since we were interested in the low-level sensorimotor properties, we focused on the movement time variability (to learn how the reaction time relates to smartphone behavior see Supplementary Note). In our multiple linear regression analysis of movement time variability, we treated the number of daily touches on the Social and Non-social Apps (all Log_10_-normalized), gender (dummy variable, female = 1), typical rate of touchscreen touches, and the number of Apps used during the recording period, as explanatory variables. The full regression model was highly significant (*R*^2^ = 0.45, *f* (6,48) = 6.5, *p* = 4.43 × 10^−5^, robust multiple linear regression; for variation inflation factors see Supplementary Fig. 2). The measured variability was inversely proportional to all but two of the explanatory variables (Fig. 1c, d, e, f). First and surprisingly, the higher number of touches on Social Apps led to increased movement time variability (Fig. 1f, for verification using randomly labeled Apps see Supplementary Fig. 3). Second, the gender was not significantly associated with the variability [*t*(1,48) = –0.90, *p* = 0.37, *Main Effect* = −1.06]. By leveraging the day-to-day variation in the rate of touchscreen interactions, we found an inverted V-shaped dynamic such that the relationship strength was the strongest when we used the data that was recorded 10.5 days prior to the lab measure (*f* = 33.59, *p* = 0.02, *t*-test compared to the distribution of *f*-values, Fig. 1c, insert).

**Fig. 2:**
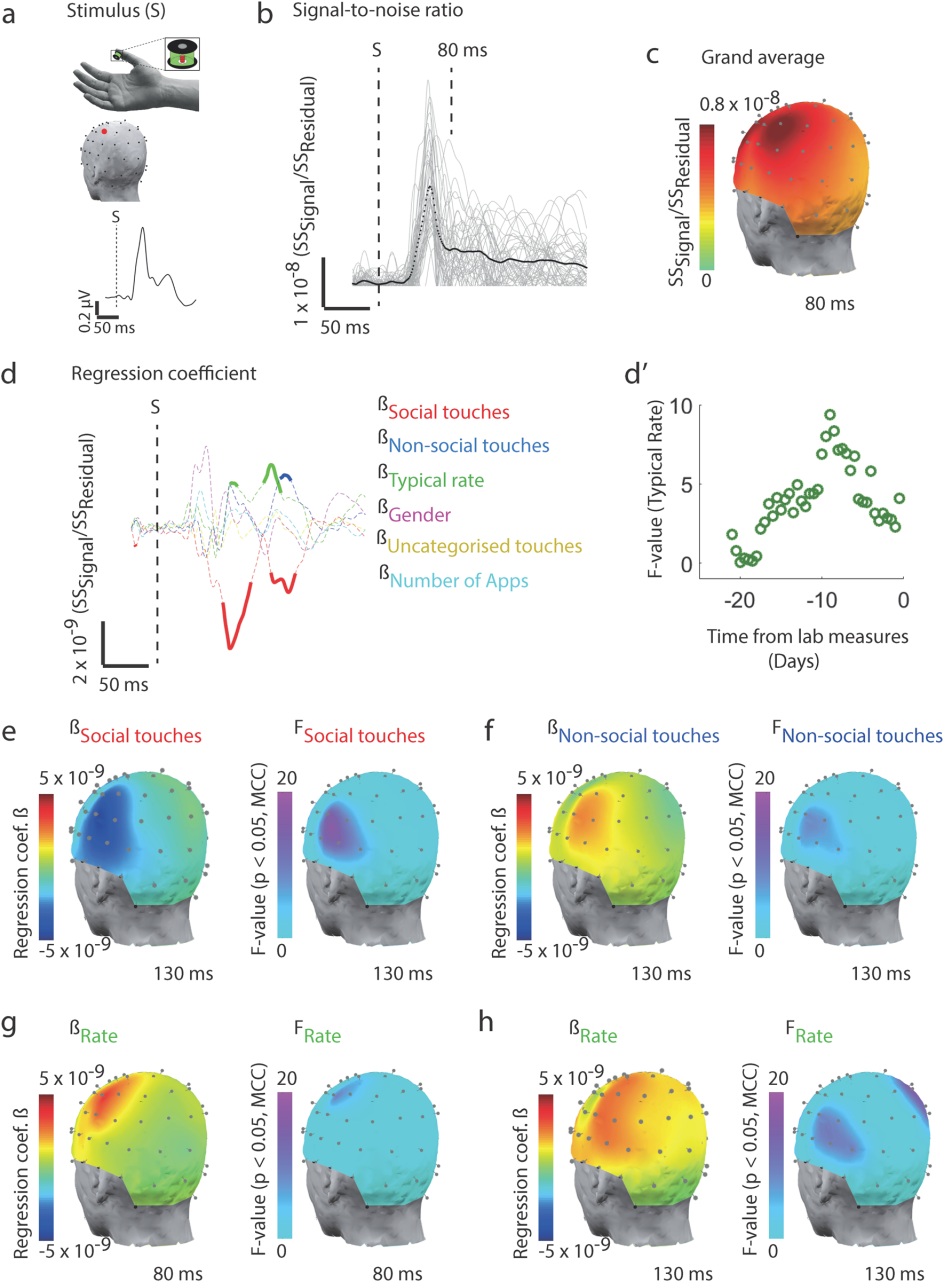
Early cortical somatosensory processing reflects the history of Social App usage. **a** We estimated the signal-to-noise ratio in the cortical responses upon a brief tactile stimulus presented to the right thumb tip, the hand was in a resting position during the recording. The head plot shows the electrode location with the best response (red). **b** Putative signal-to-noise ratio (SNR) at the electrode (SS, sum of squares). Individual volunteers (gray lines) and population mean (black). **c** Scalp map of SNR at 80 ms post stimulation. **d** Event-related coefficients with the SNR as dependent variable and touchscreen parameters based on the entire 21 days of recording as explanatory variables. Statistically significant coefficients (thickened lines, *p* < 0.05, corrected for multiple comparisons, ANOVA). **d’** The strength of the relationship with the typical rate varies as a function of the data collection period [assessed by using a 72 h sliding window, sliding with 12 h steps]. The data collected 9 days prior to the experiment yielded the strongest correlation. **e–h** Head plots of the coefficients and the corresponding variables. The statistics included all electrodes and time points, but select time points are shown to illustrate the statistical maps of the significant relationships

We measured the cortical potentials in response to tactile stimulation of the thumb using electroencephalography (EEG). The EEG signals were noisy at a single trial level and an averaging method across several trials revealed an event-related potential for the electrodes positioned above the somatosensory cortex (Fig. 2a, b, c).^9^ We used the ratio between the average response and a trial-to-trial deviation from the average as a measure of putative signal-to-noise ratio (SNR) across each of the electrodes. SNR in cortical somatosensory signals has not been previously addressed in the context of use-dependent plasticity in the elite performers. Albeit tangential from the topic of use-dependent plasticity, it has been recently explored in autism and autistic individuals show lower SNR in early cortical sensory processing—across all modalities including the somatosensory system.^10^ We found that the significant relationships with the rate, the social, and the non-social touches were largely restricted to the SNR derived from the electrodes above the contralateral sensorimotor cortex (Fig. 2d, e, f). The higher the rate on the touchscreen the higher was the SNR between 70 and 100 ms, and then again between 125 and 150 ms. In contrast, the number of social touches was inversely correlated with the SNR at time points between 70 and 100 ms, and then again between 125 and 150 ms (Fig. 2d, for verification using randomly labeled Apps see Supplementary Fig. 4). These latencies implicate the primary and secondary somatosensory, and frontal cortices.^11,12^ The pattern in the EEG measures—increased SNR associated with the rate of touchscreen use and the amount of non-social activity, and the decreased ratio associated with the amount of social activity—was consistent with the pattern of results from trial-to-trial motor variability. Similar to the motor variability, the relationship strength to the rate of touchscreen interactions showed an inverted V-shaped dynamic such that the correlation was the strongest when using the smartphone data that was collected 9 days prior to the laboratory measures (*f* = 13.98, *p* = 0.02, *t*-test compared to the distribution of *f*-values, Fig. 2d’).

## Discussion

We combined the in-depth quantification of day-to-day actions and the reduced laboratory experiments to assess how the unconstrained smartphone behavior reflect in sensorimotor computations. Our findings support the view that the low-level sensorimotor processes do not simply translate the current sensory information for motor control but they are deeply connected to the rich behavioral history. Furthermore, the cortex may be fine-tuned to day-to-day smartphone behavior such that some behavioral attributes lead to reduced neuronal noise while others increase the same. Taken together with the previous work on elite athletes and musicians, our study suggests that the low-level sensorimotor processes are capable of maintaining or accessing the impressions of a wide range of activities, from the specialized musical skills to the historical details of the spontaneous actions on the smartphone.

This study was not designed to investigate the causal mechanisms linking smartphone behavior to the laboratory measures but to quantitatively explore how the behavioral details are correlated to neuronal processes. Moreover, the laboratory measures were limited to the sensorimotor system but other systems as in the neuronal networks for reward and attention may play an important role—perhaps underlying the distinct correlates of social activity found here.^13,14^ According to an emerging idea digital behavior can be used to understand inter-individual psychological differences.^15^ This study extends that idea to neurological measures and suggests that the rich digital history captured on smartphones may help explain the differences in elementary neuronal properties.

## Methods

This study was conducted on 57 adults who were between 18 to 35 years (26 females). The day-to-day touchscreen touches was unobtrusively quantified across all Apps by using TapCounter (QuantActions GmbH, Lausanne, Switzerland). The laboratory experiments were conducted after 21 days of smartphone monitoring. Informed consent was obtained from all of the participants and the study was approved by the cantonal ethical commissions of Zurich and Vaud in accordance to the Swiss law on human experimentation. For detailed description of all of the procedures and statistical assessments used in this study see Supplementary Methods.

### Preprint server reference

An earlier version of this manuscript is deposited here: Ghosh A., Balerna M. 2016. Neuronal control of the fingertips is socially configured in touchscreen smartphone users. bioRxiv doi:10.1101/064485.

### Data availability

The data sets generated during and/or analyzed during the current study are available from the corresponding author on reasonable request.

## Electronic supplementary material

Supplementary Figure 1

Supplementary Figure 2

Supplementary Figure 3

Supplementary Figure 4

Supplementary Methods

Supplementary Note

## Electronic supplementary material

**Supplementary information** accompanies the paper on the *npj Digital Medicine* website (10.1038/s41746-017-0011-3).
