# Supplementary material for: The details of past actions on a smartphone touchscreen are reflected by intrinsic sensorimotor dynamics
Source: NPJ Digit Med. 2018 Mar 7;1:4. doi: 10.1038/s41746-017-0011-3 (PMC6548339; doi:10.1038/s41746-017-0011-3)
Supplement: Supplementary file 1 — Supplementary Figure 1 [file 41746_2017_11_MOESM1_ESM.pdf]

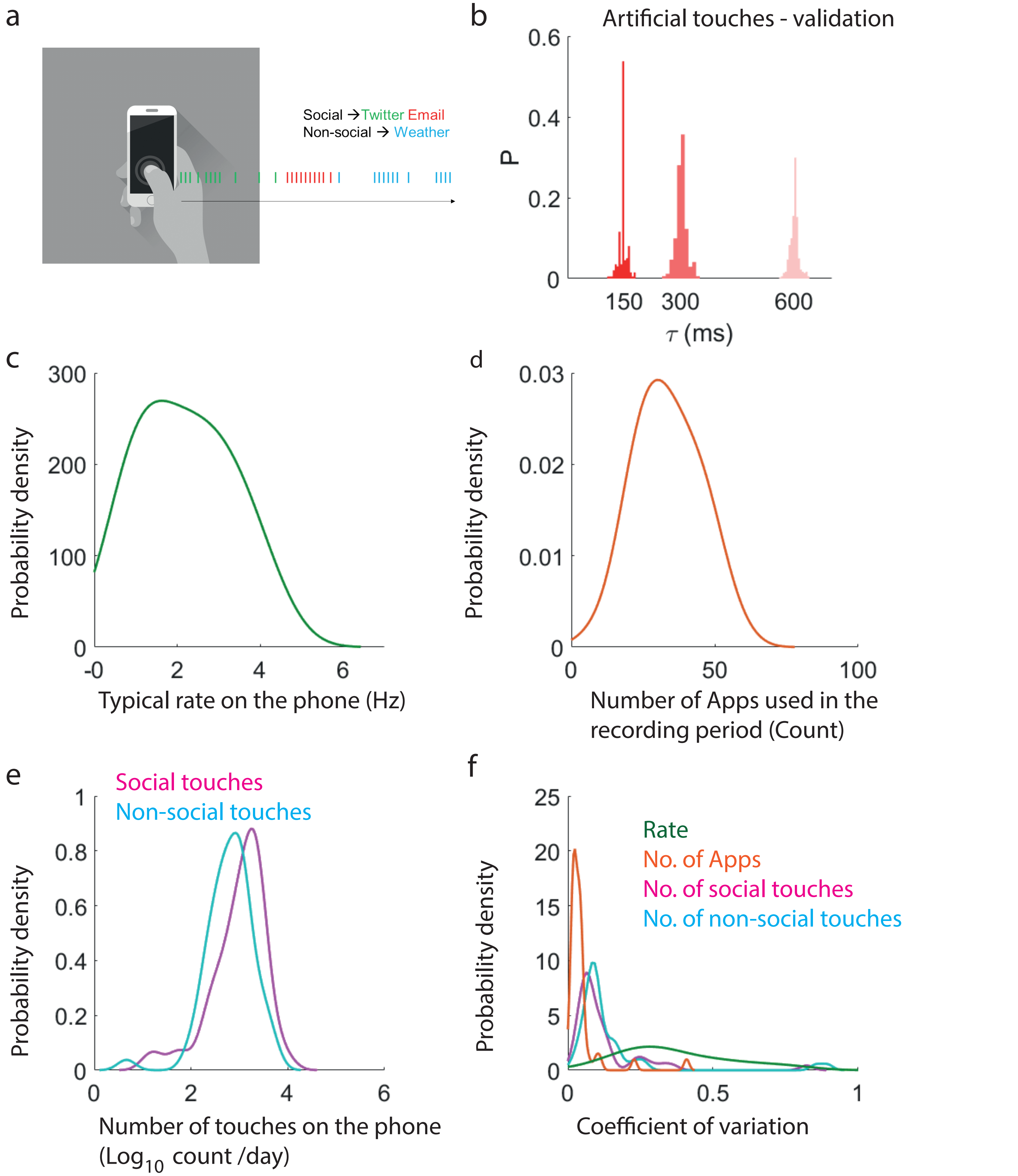

Figure S1: Non-obtrusive touchscreen recording reveals the day-to-day behavior on the smartphone. (a) We recorded the timing of all of the touchscreen touches along with the label of the App in use. (b) The temporal resolution of the recording was validated using artificial touches generated on the phone separated by 150, 300 and 600 ms. (c-e) There was substantial inter-individual variability for all of the examined parameters according to the kernel smoothed probability density plots, (c) the typical rate of touchscreen use, (d) the number Apps used in the recording period – used as a proxy for behavioral diversity & (e) the number of social & non-social touches generated on the phone. (f) Based on the day-to-day variations, we estimated the coefficient of variation in each volunteer. The day-to-day variation [assessed by using a 72 h sliding window, sliding with 12 h steps] was the highest for the typical rate of phone use compared to the other parameters.
