# Supplementary material for: The details of past actions on a smartphone touchscreen are reflected by intrinsic sensorimotor dynamics
Source: NPJ Digit Med. 2018 Mar 7;1:4. doi: 10.1038/s41746-017-0011-3 (PMC6548339; doi:10.1038/s41746-017-0011-3)
Supplement: Supplementary file 2 — Supplementary Figure 2 [file 41746_2017_11_MOESM2_ESM.pdf]

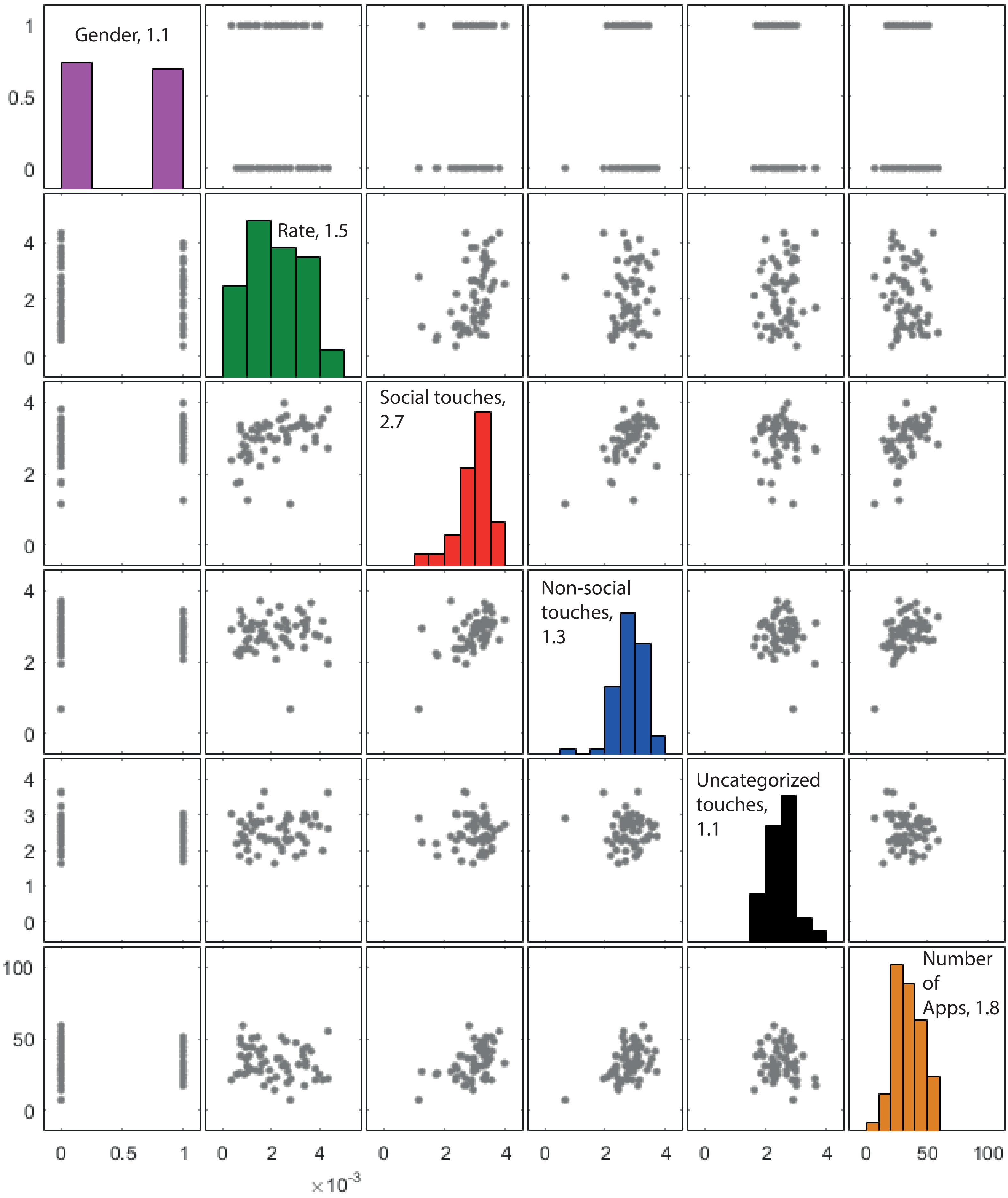

Figure S2: The plot matrix of the explanatory variables and the corresponding variation inflation factors.
