# Supplementary material for: The details of past actions on a smartphone touchscreen are reflected by intrinsic sensorimotor dynamics
Source: NPJ Digit Med. 2018 Mar 7;1:4. doi: 10.1038/s41746-017-0011-3 (PMC6548339; doi:10.1038/s41746-017-0011-3)
Supplement: Supplementary file 4 — Supplementary Figure 4 [file 41746_2017_11_MOESM4_ESM.pdf]

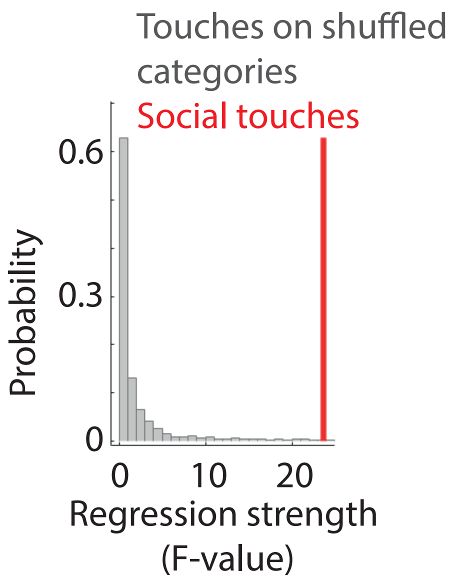

Figure S4: At the chosen electrode and at 80 ms, the distribution of the relationship strength based on randomly categorized Apps (10^4 iterations) in comparison to the relationship uncovered for social touches.
