# Supplementary material for: The details of past actions on a smartphone touchscreen are reflected by intrinsic sensorimotor dynamics
Source: NPJ Digit Med. 2018 Mar 7;1:4. doi: 10.1038/s41746-017-0011-3 (PMC6548339; doi:10.1038/s41746-017-0011-3)
Supplement: Supplementary file 5 — Supplementary Methods [file 41746_2017_11_MOESM5_ESM.pdf]

The details of past actions on a smartphone touchscreen are reflected by intrinsic sensorimotor dynamics

Myriam Balerna and Arko Ghosh

Supplementary Methods

Subjects

Participants (n = 57) were recruited using campus-wide announcements at the University of Zurich and ETH Zurich between December 2014 and August 2015. The sample consisted of subjects within a narrow age group [26 females; 23 (20th percentile) to 28 (80th percentile) years old]. The age at which the volunteers reportedly began using the phone was also narrowly distributed [19 (20th percentile) to 25 (80th percentile) years old]. Previous reports on inter-individual variability in cortical somatosensory signal-to-noise ratio, touchscreen use-dependent plasticity and use-dependent reduction in sensorimotor variability employed a sample size between 15 – 28 [1–4]. We hypothesized a weaker impact of the social touches on the touchscreen than the explanatory variables studied before, i.e., deliberate laboratory practice, touchscreen use in general and the presence of autism spectrum disorder. Therefore, we doubled the sample size and employed more regression parameters than the previous studies to increase the sensitivity of our analysis. All experimental procedures were conducted according to the Swiss Human Research Act approved by the cantons of Zurich and Vaud. The procedures also conformed to the Declaration of Helsinki. All of the experimental protocols were approved by the ethical committees of cantons of Zurich and Vaud. The volunteers provided written and informed consent before participating in the study. Right-handedness, and ownership of a non-shared touchscreen smartphone were pre-requisites for participation. The handedness was further verified by a questionnaire [5].

Smartphone data collection and analysis

A custom-designed background App was installed on the volunteers' smartphones to quantify the touchscreen behavior (App available through QuantActions GmbH, Lausanne, Switzerland). Briefly, the App recorded the timestamps of touchscreen events and the label of

the App on the foreground. The App recorded the touchscreen events with an interquartile error range of 5 ms. Data were stored locally and transmitted by the user at the end of the observation period via secure email. Smartphone data were processed using custom written scripts on MATLAB (MathWorks, Natik, USA). The number of touches on each App category ("Social", "Non-social", or "Uncategorized", see ***figure*** and list below for a sample) was divided by the length of the recording period to determine the number of touches per day. Apps that functioned to enable social interactions between a circle of friends or acquaintances were labeled as "Social" and Apps that clearly did not feature this functionality were labeled as "Non-social". Apps whose label was poorly registered by the operating system, untraceable on Google Play, or that contained both social and non-social properties, e.g., gaming Apps with social messaging, were labeled as "Uncategorized". The touches that were separated by less than 50 ms were eliminated from further analysis as considered an artefact. The rate of touchscreen events was determined as $\frac{1}{\text{Median inter-touch interval}}$. A recording period of up to 21 days was used for the main regression analysis. The number of Apps that were used over the recording period was counted.

Figure. Sample of the Apps in the database to illustrate the categorization used in the study.

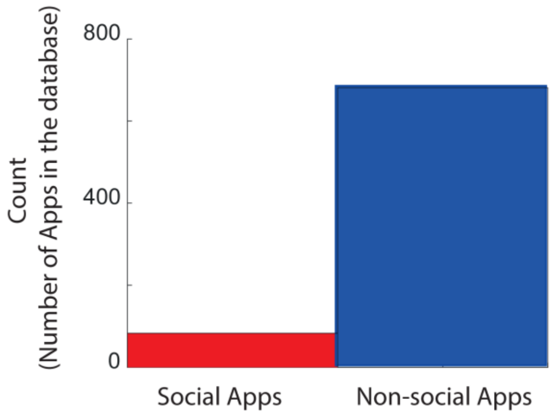

Sample of Social Apps in the database

'BLINQ'
'E-Mail'
'Facebook'
'Gmail'
'Hangouts'
'Instagram'
'Jodel'
'Jumblo'
'Messenger'
'SayHi'
'Skype'
'Snapchat'
'Tinder'
'Tumblr'
'Twitter'
'Viber'
'WeChat'
'WhatsApp'

Sample of Non-social Apps in the database

'AccuWeather'
'Adobe'
'Alarm/Clock'
'Amazon'
'BBC'
'Calculator'
'Calendar'
'Camera'
'Cleartrip'
'Climbing'
'Clock'
'Currency'
'Dictionary'
'Dropbox'
'E-Radio'
'EBook'
'Economist'
'Fitbit'
'GPS'
'Google'
'Heart'
'IKEA'
'IMDb'
'Lifelog'
'Migros'
'NBA'
'NYTimes'
'Notes'
'Reuters'
'SBB'
'TIME'
'TripAdvisor'
'Weather'
'Wikipedia'
'Zalando'
'iQuran'

Simple reaction time task and analysis

Volunteers responded to a brief (10 ms) tactile pulse by depressing and releasing a button mounted on a micro switch. The tactile pulse was presented by using a computer-controlled solenoid tactile stimulator (Heijo Research Electronics, London, UK). The stimulating magnetic rod (2 mm in diameter) generated a supra-threshold 2-mN contact. The finger stimulated and used to perform depressing and releasing actions was the thumb. The micro switch (extracted from RX-300 optical mouse, Logitech, Lausanne, Switzerland) was operated by press-downwards and release-upwards movements of the thumb. The mechanical parts for the release-upwards movement malfunctioned in two volunteers and in one of the two

the parts for press-downwards malfunctioned as well, therefore the corresponding data were eliminated from further analysis.

The task was repeated 500 times within an experimental session, with 2 min break in the middle of the session. The pulses were delivered with 3 ± 1 s gap and the button presses generated analogue signals that were digitized at 1 kHz. The reaction time (time taken to execute button depression) and movement time (time taken to execute button release) were fitted with three ex-Gaussian parameters. This form of fitting separates skewed reaction time data into a Gaussian region and an exponential region. Mean of the Gaussian region was captured by parameter μ, and variability of the Gaussian region by parameter σ. The exponent τ captured unusually slow responses. The parameters were estimated using previously described MATLAB scripts [6].

EEG data acquisition and analysis

A subset of volunteers (randomly chosen, n = 43) participated in EEG experiments. The volunteers were seated upright for the EEG and the right, stimulated, hand was concealed by using a textile baffle. Computer-controlled solenoid tactile stimulator was attached to the right thumb tip. To ease the tedium of the hours-long measurements required for gathering the tactile evoked potentials data (SSEPs), volunteers were allowed to view a movie (David Attenborough's Africa series); white noise, played to mask the sound generated by the stimulator, was mixed with the movie soundtrack and delivered through headphones. Due to technical malfunction during the measurements, one volunteer was eliminated from further analysis. The number of trials was set to 1000 and the stimuli were separated by 2–4 s. A non-alcoholic and caffeine-free drink break was offered every 10 min, for a maximum of 10 min. To record the EEG signals, 64 electrodes were used (62 equidistant scalp electrodes and two ocular ones), against a vertex reference (EasyCap, Herrsching, Germany), as previously reported [4]. The electrode locations were digitized in a 3D nasion-ear coordinate frame (ANT Neuro and Xensor software, Netherlands) for a representative volunteer. The signals were recorded and digitized by BrainAmp (Brain Products GmbH, Gilching, Germany) at 1 kHz. Offline data processing was accomplished using EEGLAB, a toolbox designed for EEG analysis on MATLAB [7]. The data were referenced to the average of all scalp electrodes and band-pass filtered between 1 and 80 Hz. "Epoched" trials over 80 μV were eliminated to remove large signal fluctuations, e.g., from eye blinks. The data were further processed using independent component analysis. Components dominated by eye movements and other measurement artifacts were eliminated by using the EEGLAB plug-in SASICA [8]. The signal-to-noise ratio was estimated using the linear modeling toolbox LIMO EEG (EEGLAB plug-in) [9]. In this toolbox, R^2 values were estimated for each volunteer based on single trials, as a sum of squares of the putative signal divided by the sum of squares of the residuals. Essentially, the

predominant notion in the sensory evoked potential research field is that the average over multiple trials extracts a signal that is otherwise hidden in the measurement noise and background neuronal processes [10]. The signal-to-noise ratio in this case captures how well the estimated mean (putative signal) represents the data. To normalize the data across the sampled population, the square root of the putative signal-to-noise ratio was used for subsequent analyses using multiple linear regression.

Correlational statistics

All analyses involving the reaction and movement times were conducted by robust–bi-square–multiple linear regression analysis (implemented in MATLAB). The fitted model was evaluated using ANOVA with a level of significance set at p = 0.05. The following main regression equation was used:

(Equation. 1)

$$Y = \beta_0 + \beta_1 X_{Touches\ on\ Non-social\ Apps} + \beta_2 X_{Touches\ on\ Social\ Apps} + \beta_3 X_{Touches\ on\ Uncategorized\ Apps} + \beta_4 X_{Rate\ of\ touchscreen\ touches} + \beta_5 X_{Number\ of\ Apps\ on\ the\ touchscreen} + \beta_6 X_{Gender\ (female=1)}$$

Where Y took the form of $Y_{Movement\ time\ variability}$ or $Y_{Reaction\ time\ variability}$, or $Y_{Somatosensory\ putative\ signal-to-noise\ ratio}$. $\beta_{1\ to\ 6}$ comprised regression coefficients estimated by robust regression, and β_0 the intercept. The explanatory variables quantifying the touchscreen behavior were based on 21 days of recording made prior to the laboratory measures.

As a control, we repeated the analysis with shuffled App categories. Essentially, for the original analysis, the Apps were labeled as “Social”, “Non-social”, and “Uncategorized”

according to a fixed criterion, i.e., Social Apps were those that enabled the communication of a message or an opinion to a circle of friends or acquaintances. The list of all Apps in the database and their classifications were randomly shuffled (10^4 iterations). These shuffled lists were then used to estimate the number of touches in each of the action categories. Note that the total number of Apps in each category was constant during shuffling. To analyze the time-dependent structure of regression parameters associated with the rate of touchscreen touches, we used the following approach. The parameter X_{Rate} was re-estimated with 12-h steps and 72-h windows while other parameters remained unchanged.

Plots for displaying multiple linear regression results in two dimensions (adjusted response plots) were generated using a built-in MATLAB function (plotAdjustedResponse). Formulation of this plotting method and its advantages are described elsewhere [11].

The EEG data were correlated with touchscreen parameters using robust regression, the iterative least squares method (implemented in LIMO EEG). The correlation coefficients were estimated across all electrodes and for the time period from –30 to 200 ms relative to the stimulation onset. The regression statistics were corrected for multiple comparisons by using 1000 bootstraps and spatiotemporal clustering, as implemented in LIMO EEG.

References

1. Dinstein, I. *et al.* Unreliable Evoked Responses in Autism. *Neuron* **75,** 981–991 (2012).
2. Cohen, R. G. & Sternad, D. Variability In Motor Learning: Relocating, Channeling and Reducing Noise. *Exp. Brain Res. Exp. Hirnforsch. Exp. Cerebrale* **193,** 69–83 (2009).
3. Gentner, D. R. The acquisition of typewriting skill. *Acta Psychol. (Amst.)* **54,** 233–248 (1983).
4. Gindrat, A.-D., Chytiris, M., Balerna, M., Rouiller, E. M. & Ghosh, A. Use-Dependent Cortical Processing from Fingertips in Touchscreen Phone Users. *Curr. Biol.* **25,** 109–116 (2015).
5. Oldfield, R. C. The assessment and analysis of handedness: The Edinburgh inventory. *Neuropsychologia* **9,** 97–113 (1971).
6. Lacouture, Y. & Cousineau, D. How to use MATLAB to fit the ex-Gaussian and other probability functions to a distribution of response times. *Tutor. Quant. Methods Psychol.* **4,** 35–45 (2008).
7. Delorme, A. & Makeig, S. EEGLAB: an open source toolbox for analysis of single-trial EEG dynamics including independent component analysis. *J. Neurosci. Methods* **134,** 9–21 (2004).
8. Chaumon, M., Bishop, D. V. M. & Busch, N. A. A practical guide to the selection of independent components of the electroencephalogram for artifact correction. *J. Neurosci. Methods* **250,** 47–63 (2015).
9. Pernet, C. R., Chauveau, N., Gaspar, C. & Rousselet, G. A. LIMO EEG: A Toolbox for Hierarchical LInear MOdeling of ElectroEncephaloGraphic Data. *Comput. Intell. Neurosci.* **2011,** e831409 (2011).
10. Makeig, S., Debener, S., Onton, J. & Delorme, A. Mining event-related brain dynamics. *Trends Cogn. Sci.* **8,** 204–210 (2004).
11. DuMouchel, W. Graphical representation of main effects and interaction effects in a polynomial regression on several predictors. in *Proc. 20th Symp. on the Interface: Computer Science and Statistics* 127–134 (1988).
