# Supplementary material for: The details of past actions on a smartphone touchscreen are reflected by intrinsic sensorimotor dynamics
Source: NPJ Digit Med. 2018 Mar 7;1:4. doi: 10.1038/s41746-017-0011-3 (PMC6548339; doi:10.1038/s41746-017-0011-3)
Supplement: Supplementary file 6 — Supplementary Note [file 41746_2017_11_MOESM6_ESM.pdf]

The details of past actions on a smartphone touchscreen are reflected by intrinsic sensorimotor dynamics

Myriam Balerna and Arko Ghosh

Supplementary Note

Fingers used on the touchscreen

To assess the fingers mostly used on the phone, we used a conventional approach. By using a pictorial survey the volunteers ranked the preferred fingers used on the smartphone. Confirming previous findings for smartphone usage, the thumb was ranked by 73% of the volunteers as most preferred on the touchscreen; 16% preferred the index finger; and 10% preferentially used both the thumb and the index finger [1–3]. Only one volunteer preferred his middle finger on the phone to all of the other fingers.

Only social touches & gender reflect on reaction time variability

We explored the variability of higher cognitive levels captured by the reaction time. For the reaction time variability, the full regression model – including the rate of touchscreen use, the number of Apps used, the number of touches on Social Apps, the number of touches on Non-Social Apps, and the gender of the participant – was significant but weak [$R^2 = 0.26$, $f(6,49) = 2.86$, $p = 0.02$, robust linear regression]. Similarly to the results for movement time variability, we observed that a higher number of social touches was associated with greater reaction time variability [$t(1,49) = 2.72$, $p = 0.009$, comparing the coefficient obtained from movement time variability vs. reaction time variability, $p = 0.587$]. The only other explanatory variable that significantly contributed to the regression model was the participant gender, such that the females showed less variability [$t(1,49) = –3.25$, $p = 0.0002$] than the males. Since the reaction and movement times measure different aspects of cognition, taken together, they suggested that the putative impact of social touches is not restrained to the lower-levels of sensorimotor cognition.

References

1. Gindrat, A.-D., Chytiris, M., Balerna, M., Rouiller, E. M. & Ghosh, A. Use-Dependent Cortical Processing from Fingertips in Touchscreen Phone Users. *Curr. Biol.* **25,** 109–116 (2015).
2. Xiong, J. & Muraki, S. An ergonomics study of thumb movements on smartphone touch screen. *Ergonomics* **57,** 943–955 (2014).
3. Bergstrom-Lehtovirta, J. & Oulasvirta, A. Modeling the Functional Area of the Thumb on Mobile Touchscreen Surfaces. in *Proceedings of the 32Nd Annual ACM Conference on Human Factors in Computing Systems* 1991–2000 (ACM, 2014). doi:10.1145/2556288.2557354
